# Infrequent occurrence of *TET1, TET3*, and *ASXL2* mutations in myelodysplastic/myeloproliferative neoplasms

**DOI:** 10.1038/s41408-018-0057-8

**Published:** 2018-03-12

**Authors:** Terra L. Lasho, Rangit Vallapureddy, Christy M. Finke, Abhishek Mangaonkar, Naseema Gangat, Rhett Ketterling, Ayalew Tefferi, Mrinal M. Patnaik

**Affiliations:** 1 Division of Hematology, Department of Medicine, Mayo Clinic, Rochester, MN USA; 2 Department of Laboratory Medicine and Pathology, Mayo Clinic, Rochester, MN USA

Ten Eleven Translocation (TET) proteins are a family of dioxygenases (TET1, TET2, and TET3) that catalyze the oxidation of 5-methyl-cytosine (5mC) to 5-hydroxymehylcytosine (5hmC), 5-formlycytosine (5fC), and 5-carboxylcytosine (5caC)^1^. Mutations involving *TET2* (4q24) have widely been reported in the context of age-related clonal hematopoiesis (~10% >80 years of age)^2^, and hematological malignancies such as myelodysplastic syndromes (MDS 5–20%), myeloproliferative neoplasms (MPN~15%), chronic myelomonocytic leukemia (CMML ~60%), acute myeloid leukemia (AML 8–30%), and T and B cell lymphoproliferative disorders^3–5^. In CMML, thus far, clonal *TET2* mutations in the absence of clonal *ASXL1* mutations (*ASXL1*wt/*TET2*mt) have been associated with favorable outcomes^6^. Conversely, mutations in *TET1* (10q21.3) and *TET3* (2p13.1) are extremely infrequent with a large study of 408 MPN, CMML, and AML patients demonstrating no identifiable mutations in these genes^3^. In a recent study, whole exome sequencing was performed in 49 CMML patients resulting in the detection of two loss-of-function, subclonal, *TET3* mutations (R148H and S1708fs), both in patients with co-existing *TET2* mutations^7^. *ASXL2* (additional sex combs-like; 2p23.3) mutations were recently described in adult and pediatric patients with t(8;21)/core binding factor AML (*RUNX1–RUNX1T1*) (~20%) and were associated with a higher cumulative incidence of relapse^8^. In MDS/MPN overlap syndromes including CMML, thus far, the frequency and prognostic impact of *ASXL2* mutations remain unknown. We carried out this study to estimate the frequency and clinical correlates of *TET1, TET3*, and *ASXL2* mutations in patients with MDS/MPN overlap syndromes.

Eighty three patients meeting the 2016 World Health Organization (WHO) criteria for CMML (*n* = 30) and MDS/MPN-Unclassifiable (MDS/MPN-U, *n* = 47) were included in the study^9^. The median age was 73 years (range, 18–89 years) and 66% were male. All patients had bone marrow (BM) biopsies and cytogenetic studies performed at diagnosis. Target capture-based next generation sequencing (NGS) was carried out on diagnostic BM DNA from all 83 patients for the complete coding regions of the following 42 genes: *TET1*, *TET2,TET3, DNMT3A, IDH1, IDH2*, *ASXL1, ASXL2, ATM, EED, EZH2, JARID2, SUZ12*, *BCOR, BCORL1, STAG2, GATA2, TERC, TERT, SRSF2, SF3B1, ZRSR2, U2AF1*, *PTPN11,PHF6, Tp53, SH2B3, RUNX1, CBL, NRAS, KRAS, JAK2, CSF3R, FLT3, KIT, CALR, MPL*, *NPM1, CEBPA, IKZF1, ETNK1*, and *SETBP1* by previously described methods^6^. Paired-end indexed libraries were prepared from individual patient DNA using the NEBNext Ultra Library prep protocol on the Agilent Bravo liquid handler. Capture libraries were assembled according to Nimblegen standard library protocol. Base-calling was performed using Illumina’s RTA version 1.17.21.3. Genome_GPS v4.0.1 (formerly named as TREAT) was employed to analyze the data^10^. Specific variants were included if they were cited by the Catalog of Somatic Mutations in Cancer database (COSMIC, http://cancer.sanger.ac.uk) and/or if they were found at less than 0.1% by the Exome Aggregation Consortium (ExAC, Broad Institute, Cambridge, MA) and not associated with a COSMIC identifier. Previously annotated single-nucleotide polymorphisms (http//www.hapmap.org) in these genes were excluded. For *ASXL1*, only frameshift and nonsense mutations were considered pathogenic^11^.

**Fig. 1:**
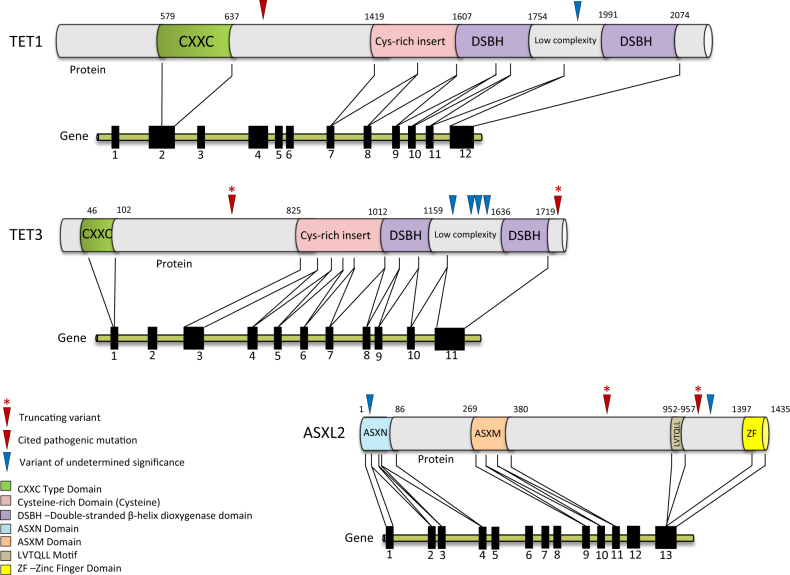
Domain architecture of *TET1, TET3*, and *ASXL2* with observed gene mutations and variants of unclear significance

Overall, we observed seven patients (5 CMML [17%] and 2 MDS/MPN-U [4%]) with mutations and/or VUS involving *TET1* and *TET3* (Table 1 and Fig. 1). All cases were without concurrent *TET2* mutations. Of these, loss-of-function *TET1* and *TET3* mutations were identified in two patients, both with a morphological diagnosis of CMML. Patient one is a 66-year-old male with CMML-0 and normal cytogenetics (Mayo Molecular Model/MMM—intermediate-1 risk) who had presented with monocytosis and thrombocytopenia^11^. BM NGS analysis revealed a *TET3*K1760del (48%—variant allele frequency), with an additional *PHF6*F172Lfs*46 (88%) mutation. He is being treated with 5-azacitidine and at last follow up (15.5 months) remains in a morphological complete remission (CR) after 10 cycles of therapy. Patient two was an 80-year-old female with CMML-0 and normal cytogenetics (MMM—high risk), who had presented with monocytosis and circulating immature myeloid cells. BM NGS at diagnosis identified TET3Y473* (43%) with a coexisting *TET1*Q683 (46%) mutation (previously cited as pathogenic shown in Table 1), and two additional mutations: *ASXL1*G646Wfs*12 (26%) and *PTPN11*N308D (14%). She received supportive care and died within a month of diagnosis without evidence for leukemic transformation.

**Table 1:** Spectrum of *TET1, TET3* and *ASXL2* mutations and variants of unclear significance in patients with MDS/MPN overlap syndromes

Gene	Chr	Position	Nucleotide nomenclature	Protein consequence	Disease type	Alt Frac	ExAC	dbSNP	Cosmic #	Cited as somatic	Cosmic Annotated Disease Type	Exon	Phenotype Prediction	Concurrent Mutations
TET1	10	70404533	c.2047C > G	*Q683E*	CMML^b^	46%	0.08400%	rs139785845	COSM327333	yes	ALL^1^, Sezary Syndrome^2^	4	MODERATE	*TET3*Y473^a^ (43%) *ASXL1*G646Wfs^a^12 (26%) *PTPN11*N308D (14%)
		70450700	c.5540G > T	G1847V	MDS/MPNu	52%	n/a	n/a	n/a			12	MODERATE	*SRSF2*P95R (53%) *NRAS*G13D (41%)
TET3	2	74274463	c.1419C > A	*Y473* ^*a*^	CMML^b^	43%	n/a	n/a	n/a			3	HIGH	*TET1*Q683E (46%) *ASXL1*G646Wfs^a^12 (26%) *PTPN11*N308D (14%)
		74329187	c.5278_5280del	*K1760del*	CMML	48%	0.02900%	rs564392898	n/a			11	MODERATE	*PHF6*F172Lfs^a^46 (88%)
		74327798	c.3883G > A	V1295I	CMML	51%	0.04400%	rs199849765	n/a			11	MODERATE	*CEBPA*H195_P196dup (57%) *ATM*L1111P (51%) *ASXL1*L775^a^ (49%) *JARID2*R767K (46%)
		74327893	c.3980_3981insACTGAG	N1326_S1327insRL	CMML^a^	41%	0.00860%	rs768310475	n/a			11	MODERATE	*SRSF2*P95T (45%) *TET3*L1328P (42%)
		74327898	c.3983T > C	L1328P	CMML^a^	42%	0.00860%	rs767538752	n/a			11	MODERATE	*SRSF2*P95T (45%) *TET3*N1326_S1327insRL (41%)
		74328177	c.4262C > G	P1421R	CMML	51%	0.00940%	rs745953793	n/a			11	MODERATE	*SH2B3*R140H (58%) *JARID2*P1229L (52%) *NRAS*Q61K (47%) *RUNX1*G199W (21%)
		74329152	c.5237G > T	W1746L	MDS/MPNu	49%	0.06500%	rs190925009	n/a			11	MODERATE	*SRSF2*P95L (49%) *ASXL1*P808H (49%) *JAK2*V617F (49%)
ASXL2	2	25966302	c.2902_2903dupCT	*P969Cfs* ^a^ *10*	MDS/MPNu	22%	n/a	.	n/a			13	HIGH	*SRSF2*P95_R102del (15%) *RUNX1*R237K (38%)
		25967305	c.1901C > A_p.Ser634X	*S634* ^a^	MDS/MPNu	20%	n/a	.	n/a			13	HIGH	*SRSF2*R94dup (39%)
		25965934	c.3272 C>T_p.Ala1091Val	A1091V	CMML	51%	0.01300%	rs781151810	n/a			13	MODERATE	ZRSR2 c.400-2A > G (92%) MPLV368L (49%) ASXL1G646Wfs^a^12 (44%) SETBP1D868N (43%) RUNX1T246Hfs^a^15 (35%)
		26101079	c.13G > A_p.Gly5Arg	G5R	MDS/MPNu	51%	0.01200%	rs371056638	n/a			1	MODERATE	EZH2 c.1411-1G > A (91%) *ASXL1*R417^a^ (45%) *JARID2*R326C (48%) *SUZ12*N263H (47%)

Values in italic denote cited pathogenic mutation or variants which truncate the protein

^a^ Mutations in same patient (*TET3*N1326_S1327insRL and *TET3*L1328P)

^b^ Mutations in same patient (*TET1*Q683E and *TET3*Y473^a^)

^c^ Ref. ^12^

^d^ Ref. ^13^

^e^ Ref. ^14^

^f^ Ref. ^15^

We identified *ASXL2* mutations or VUS in four patients (3 MDS/MPN-U [6%] and 1 CMML [3%]). Of these, two patients with MDN/MPN-U harbored loss-of-function *ASXL2* mutations. Patient one was a 67-year-old male with trisomy 21 who had presented with transfusion-dependent anemia and thrombocytopenia. BM NGS at diagnosis identified *ASXL2*S634* (20%) and *SRSF2*R94dup (39%). He was treated with 5-azacitdine and had no response after four cycles. He died shortly thereafter with no evidence for leukemic transformation. Patient two was a 75-year-old female with MDS/MPN-U and trisomy 8 and trisomy 9, who had presented with transfusion-dependent anemia. BM NGS at diagnosis identified *ASXL2*P969Cfs*10 (22%), *RUNX1*R237K (38%), and *SRSF2*P95_R102del (15%). She was treated with transfusional supportive care and was lost to follow-up.

Our study reveals that although uncommon, loss-of-function *TET1, TET3*, and *ASXL2* mutations can be seen in patients with MDS/MPN overlap syndromes. *TET1* and *TET3* mutations were seen exclusively in CMML, were found to coexist with each other (*TET1* and *TET3*), and occurred independent of *TET2* mutations. *ASXL2* mutations were seen in MDS/MPN-U, were associated with numerical chromosomal aberrations, and occurred independent of *ASXL1* mutations. The current study was limited by a small number of informative cases to opine on clinical correlates and survival outcomes. Studies exploring the functional redundancy of *TET1* and *TET3* mutations with TET2 activity, the impact of *TET1*, *TET3*, and *ASXL2* mutations on global and sequence-specific 5-mC and 5-hmC levels and post-translational histone modifications (H3K27me3), and the impact of these mutations on survival are currently being planned.
